# HIV prevention – Challenges in reaching Libyan women: A narrative review

**DOI:** 10.1177/17455057221080832

**Published:** 2022-02-26

**Authors:** Abier Hamidi

**Affiliations:** Faculty of Health & Social Sciences, Bournemouth University, Poole, UK

**Keywords:** HIV, Libya, MENA, prevention, women

## Abstract

**Introduction:**

The need to effectively communicate HIV/AIDS prevention messages in Libya, where HIV prevalence is relatively low yet increasing, cannot be overstressed. A review of the literature on HIV prevalence, risk factors, stigma and awareness found that there is a lack of HIV research, information and support in the country. This is particularly true regarding women, who account for 25%–30% of people living with HIV in Libya.

**Aim:**

Drawing on the various literature, this narrative review will (1) present a historical trajectory of Libyan women and their role in society and (2) identify some challenges that HIV prevention programmes face in reaching Libyan women.

**Methods:**

Medline, PubMed, Web of Science, ScienceDirect, Scopus and Cochrane Library were searched for English and Arabic language articles. Primary research studies and official reports indicating a discussion or research on HIV in Libya and Libyan women were considered. Reference lists of articles were reviewed to identify additional studies. Thirty-seven articles dating from 1987 to 2021 were selected and critically appraised.

**Results:**

There is a lack of sufficient information within the existing literature, but the gathered literature did reveal some significant insights. Factors such as limited sexual health education, inadequate medical services, social and cultural restrictions and stigma, as well as limited agency, were identified as potential barriers to women accessing crucial information on HIV.

**Conclusion:**

The article found that the HIV prevention efforts that have been carried out in Libya may be compromised as they were not designed to recognize and adhere to sociocultural norms that impact on Libyan women’s scope for choice and agency. By understanding the interplay between gender, social and structural factors in Libya, a model of better adjusted prevention and early intervention activities could be developed; a toolkit that conceptualizes the culture and that appreciates the role of a Libyan woman is changing.

## Introduction

A recent scoping review^1^ documents that although there is a lack of reliable data, HIV prevalence is increasing in Libya and growing rapidly among married women. The authors suggest that religion and culture may impact research efforts, which, in turn, increases a Libyan woman’s vulnerability to the virus.

The latest reported HIV prevalence in Libya is estimated at 0.2% (UNGASS)^2^ and that there are 10,450 registered cases,^3^ with injecting drug use identified as the principal mode of transmission. However, there is also clear evidence that cross-infection of HIV is occurring through sexual activities.^4,5^ It was found that HIV cases transmitted from sexual activities had increased steadily, reaching 40% (n = 858) between 2013 and 2017.^4^

It is speculated that marital heterosexual intercourse is the main cause of HIV transmission in Libyan women. A study on HIV-related hospitalizations found that out of the 227 people hospitalized, a majority of women living with HIV were married 57.5% (n = 23) or widowed 22.5% (n = 9). Further to this, 87.5% (n = 35) identified condom-less marital sexual relations as the route of HIV transmission.^6^

HIV cases in women are increasing; out of the 10,000 HIV cases reported in 2018,^7^ 25%–30% were women. In November 2020, the number of people with HIV (excluding the eastern part of Libya) was around 6000 of whom 35%–40% were women.^8^

Against this backdrop, this article will review published literature and identify the major challenges that Libyan women have and continue to face that could potentially increase their vulnerability to the HIV virus. The aim of this narrative review is to introduce the Libyan woman and her evolving role in Libyan society. It also explores how social and structural factors and norms constrict her access to healthcare, information and discuss implications for practice.

## Methods

Computer databases Medline, PubMed, Web of Science, ScienceDirect, Scopus and Cochrane Library were searched. This was followed by examining the reference list of key articles identified in the primary search to determine additional literature as well as performing a Google search.

Due to the limited available research on HIV prevalence in Libyan women, separate searches were performed for Libyan women and HIV in Libya. The inclusion criteria were all types of articles with title or abstract indicating a discussion or research on HIV in Libya, Libyan women and related terms, in English or Arabic. Newspaper articles were used to support and provide further details.

The exclusion criteria were articles for which full text was not available, papers that were irrelevant to the research topic and those with duplicated information.

Thirty-seven articles dating from 1987 to 2021 were selected and evaluated for this review, of which 16 were on HIV in Libya. In total, 20 papers were on Libyan women: 5 official reports, 12 journal articles, 1 newspaper article and 2 books.

## Libyan women past and present

Women constitute more than half of the Libyan population and are celebrated for playing the roles of mothers, teachers, doctors and so on. Yet, their ability to participate in any formal decision making remains insignificant and undervalued. Throughout the history of Libya, women have been placed under severe structural and cultural constraints.

Prior to independence in 1951, Libya was a poor and underdeveloped country. As with many Middle Eastern and North African (MENA) countries, it was organized along a patriarchal structure: the women worked on farms and were responsible for their families, having limited freedom outside of the home.^9^

During the Monarchy (1951–1969), it was said that Libyan women did enjoy a relative degree of freedom.^10^ They formed unions and associations and participated in influencing public opinion through their writings and votes. The progress was slow but steady, with Libyan leaders supporting women’s rights within the principles of Islam.^11^

Muammer Gaddafi and his so-called Free Officers staged a coup d’état on 1 September 1969, ending the Monarchy rule. While Gaddafi’s Libya was presented internationally as a more modern society that promoted equality between men and women, many believe that this was merely a charade.

Under Gaddafi’s dictatorship, there was a significant increase in female enrolments in the educational system, and laws were passed regulating women’s employment, including equal pay for equal work. A minimum age of 18 years old was set for marriage, and women were given equal rights for divorce.

In reality, the regime restricted Libyan women to work only in fields that Gaddafi felt suited their nature as defined by him, such as nursing, teaching, administrative and clerical work.^9^ This led to limited access to high-level employment,^12^ and the exclusion of women from the political sphere which is in direct contradiction to the equality laws. Gaddafi systematically eliminated civil rights during his dictatorship, with women who opposed the regime imprisoned or raped.^16^

17 February 2011 saw the Gaddafi regime overthrown. Libyan women played a crucial role in the success of the revolution, joining in demonstrations, actively reaching out nationally and internationally via social and traditional media. Many women provided logistical support, treated the injured and even assisted in smuggling ammunition.^13^

Libyan women were empowered in new ways. The 2011 Revolution thrusted them into roles that traditional Libyan society could not anticipate. Gender stereotypes and entrenched social taboos seemed to have faded, forcing the men to accept the women as equals but only temporarily. After the successful ousting of the regime, the women were contemptuously dismissed and were expected to return to their roles as mothers and housewives.^14^ This led to a strong women’s rights movement, with some Libyan women determined not to lose the freedom they had gained during the revolution.

Challenging the social fabric of the traditional Libyan society has resulted and continues to subject women to severe backlash; from push-back from families (as going against the cultural structure could only mean that these women are ‘improper’ and will not only tinge their reputation, but also their families), sexual harassment to threats of physical harm.^15,16^

## HIV prevention and intervention challenges

As a response to the HIV epidemic in Libya, the National Centre for Disease Control has implemented various initiatives, including training of medical staff, a mobile (Voluntary Counselling and Testing (VCT)) service, an HIV and Hepatitis Hotline as well as awareness-raising media campaigns, health education in schools and World AIDS Day celebrations.^5,17^

While the effort is apparent, Libyan women are mainly invisible in the response to HIV, as the daily structural and social challenges they face restrict their access to the interventions that are in place.

### Limited information and school-based health education

Prevention efforts around the world advocate providing accurate information on HIV/AIDS through the school-based health education system, which will, in turn, provide the knowledge and tools to prevent, treat and manage HIV and other sexually transmitted diseases (STDs).

In Libya, HIV education is limited^17^ and even then, only about half of the Libyans considered school health education as an effective medium in raising public health knowledge and influencing healthy behaviour.^18^ A recent study on HIV knowledge and perception conducted in 2015 on Libyan dental student found that their overall awareness of HIV is low. It identified that less than one-third of the students recognized injectable drug use and sexual intercourse as modes of HIV transmission.^19^

Despite the rate of female enrolment in secondary and higher education exceeding that among males,^20^ culturally women and girls are often encouraged to remain uninformed about sexual matters because of a misguided fear that it will encourage sexual activity.^21^

### Social stigma and reputation

The substantial misconceptions of HIV transmission and the perceptions of immoral practices related to HIV are the catalysts for social stigma and discriminatory behaviour. With a population of around 6.8 million, Libya is predominantly made up of well-known large families and tribes,^15^ the actions of an individual can bring collective shame to the family and to the tribe. The consequence of this is that many may avoid getting tested or seeking treatment due to the fear of bringing shame to their families as well as the social repercussions of even being suspected of acquiring HIV.^22^ Many people living with HIV in Libya have been outcasted by family and community and are unable to access healthcare, study or work.^23^

The consequences are even fiercer for girls and women as they are held to double standards related to their behaviour in public and sexual activities.^24,25^ Under Gaddafi’s rule, women and girls suspected of violating or transgressing from moral codes were detained into ‘social rehabilitation’.^14^ It is unknown whether these facilities still exist today but what is certain is that girls and women who are found to have acquired HIV are subjected to harsher stigma-related discrimination such as violence, humiliation as well as invasive virginity tests.^8^

### Safe sex negotiation and condoms

A large proportion of women living with HIV acquired the virus within a marital relationship and therefore the most effective and accessible form of protection would be to negotiate safe sex.

In a classic patriarchal household, as most Libyan households are,^26^ the woman has limited agency within the marriage to refuse sex or negotiate safe sex. The fear of the consequences of ‘disobeying’ her husband, which could include domestic violence,^24,27^ divorce and so on, is the main obstacle to negotiating the use of condoms.

Another factor is the perceived use of condoms as a form of contraception rather than a means of protection against HIV and other sexually transmitted infections. Only 156 out of 400 final year Libyan university students believed that condoms are important for the prevention of HIV transmission.^18^

### Healthcare and freedom of movement

Libyan women don’t tend to visit healthcare providers regularly, only 7% (sample of 1489) of women visit doctors annually for check-ups and 40% have never visited a gynaecologist. This is presumably due to a majority stating that the medical services in Libya are inaccessible or inadequate.^28^

Many health facilities are unable to provide adequate services due to shortage of essential medicines, medical supplies (including equipment) and staff with up-to-date training. The 2016 Service Availability and Readiness Assessment (SARA) survey by the Health Information Centre (Ministry of Health) in collaboration with the World Health Organization (WHO)^29^ country and regional offices found that significant coverage gaps exist in the provision of HIV/AIDS and sexually transmitted infections (STIs) in the country.

Additionally, the deteriorating security situation has forced Libyan women themselves and their families to restrict their movements due to the fear of being kidnapped or sexually assaulted.^30^ A survey in 2013 found that 57% of women say that they feel completely (37%) or somewhat (20%) prohibited from leaving their home without permission. 52% of men and 41% of women condone abusive behaviour against a wife should she leave the home without informing her husband.^28^

## Discussion

Having presented an overview of the social and structural factors that impact the daily lives of Libyan women, it is apparent that reaching them is a challenge. What is also indisputable is that they are defying the role that has been imposed on them.

Although Libyan women exist in an environment that is laden with many risks, they are not waiting patiently for opportunities to come but rather they are creating them. The women’s activism during and post revolution stands in sharp contrast to the stereotypical perception of Libyan women that is also shared with the West. This misconception that Libyan women should not play a role in the public sphere was evident through the media coverage of the revolution. In Gendering War and Peace,^31^ it was found that women were not visible in nearly 60% of the reports with the reporters blaming Libyan men and the culture.

Libyan women are rising to claim agency. During Gaddafi’s rule, Civil Society Organizations (CSOs) were banned; however, since the revolution, there has been a surge of women CSOs focusing on gender equality, with campaigns that challenge violence against women and their role in the public and political arena.^32^

The women’s rights movement in Libya is based on the country’s culture, history and religion,^33^ but without the cultural construct that restricts their choices and rights.^34^ Patriarchy should not be conflated with Islam and often cultural idiosyncrasies or misinterpretations are presented as religious teachings.

Libya is considered to be one of the most religiously and culturally conservative countries in the MENA region^35^ with Islam playing a substantial role in the daily lives of Libyans. It has been suggested that Islam promotes low-risk behaviour as it encourages male circumcision, prohibits sexual intercourse outside of marriage^3^ and the use of intoxicants. Be that as it may, the HIV prevalence in the country is on the rise and Libyan women are becoming increasingly vulnerable; therefore, despite Islamic teachings and cultural restrictions, some Libyans do engage in activities that lead to acquiring HIV.

The available data dictates that most Libyan women who are living with HIV have acquired the virus from their husbands, so it is essential that preventive and intervention efforts are reaching them. Nonetheless, the social, cultural and religious frameworks^36^ in the country have contributed to the under-representation of other high-risk activities that Libyan women could be engaging in, such as injection drug use,^37^ sex work or sex out of marriage.

Accordingly, any effort directed at HIV prevention needs to consider the rapidly evolving role, status and agency of Libyan women within the religious and societal restrictions of the country.

## Conclusion

Libya remains one of the least researched countries in MENA and to date there are very few publications on Libyan women, let alone in respect to HIV. This review of the literature on Libyan women’s HIV prevalence, stigma and challenges found that there is an urgent need for HIV prevention campaigns and interventions that capitalize on the social, religious and cultural structure, while appreciating that the role of a Libyan woman is continually shifting.

Efforts should be made to develop targeted campaigns with information and material that are responsive to the needs of Libyan women and disseminated through effective channels. Moreover, more attention must be paid to prevention measures for married women with the integration of HIV services into the reproductive health services in the country.

As part of the preventive strategy, women-led civil societies engaged in addressing structural interventions need to be encouraged and supported. Additionally, including Islamic leaders and teaching in HIV prevention initiatives will strengthen the efforts as well as help reduce stigma and discrimination against people living with HIV.

## References

[1] Hamidi A Regmi P van Teijlingen E. HIV epidemic in Libya: identifying gaps. J Int Assoc Provid AIDS Care 2021; 20: 23259582211053964. 34841956 10.1177/23259582211053964 PMC8640281

[2] Libyan Arab Jamahiriya UNGASS Country Progress Report 2010. UNAIDS MENA 2008, https://www.unaids.org/sites/default/files/country/documents/libyanarabjamahiriya_2010_country_progress_report_en.pdf

[3] Elhodairi MAA Mutwakil G Alamen AW , et al. HIV/AIDS in Libya. Sebha Med J 2008; 7: 36–43, https://sebhau.edu.ly/suj/paper/HIV.m.pdf

[4] Daw MA Daw AM Sifennasr NEM , et al. Spatiotemporal analysis and epidemiological characterization of the human immunodeficiency virus (HIV) in Libya within a twenty five year period: 1993-2017. AIDS Res Ther 2019; 16: 14. 31238947 10.1186/s12981-019-0228-0 PMC6591977

[5] Control NCoD. GARPR2015. Tripoli: National Centre for Disease Control, 2015.

[6] Shalaka NS Garred NA Zeglam HT , et al. Clinical profile and factors associated with mortality in hospitalized patients with HIV/AIDS: a retrospective analysis from Tripoli Medical Centre, Libya, 2013. E Mediterr Health J 2015; 21: 635–646. 10.26719/2015.21.9.635 26450860

[7] Libyan Express. 5,000 HIV infections reported in Libya in 2018. Libyan Express, https://www.libyanexpress.com/new-5000-hiv-cases-reported-in-libya-since-january-2018/

[8] Abdul Jalil R . Illness is not an accusation ‘women with HIV’ tell the story of stigma. Huna Libya, https://hunalibya.com/dammawashadda/youth-health/12527/

[9] St John RB . Libya’s gender wars: the revolution within the revolution. J North Afr Stud 2017; 22: 888.

[10] Langhi Z. Gender and state-building in Libya: towards a politics of inclusion. J North Afr Stud 2014; 19: 200–210.

[11] Azzuz IS . Libyan women: past, present, and future. In: Toperich S Mullins A (eds) A new paradigm: Perspectives on the changing Mediterranean. Washington, DC: Brookings Institution Press, 2014, pp. 149–158.

[12] Arabsheibani GR Manfor L . From Farashia to military uniform: male-female wage differentials in Libya. Econom Develop Cult Change 2002; 50: 1007.

[13] Elisabeth J-N . Gendering the Arab Spring? Rights and (in)security of Tunisian, Egyptian and Libyan women. Secur Dial 2013; 44: 393–409.

[14] HRW. Revolution for all. Women’s rights in the New Libya. Report no. 978-1-62313-0114. United States of America Human Rights Watch, 2013, Human Rights Watch, https://www.hrw.org/report/2013/05/27/revolution-all/womens-rights-new-libya

[15] Ann M . Boxed in? The women of Libya’s revolution. World Aff 2011; 174: 64–68.

[16] Lagdaf S Zoubir YH . The struggle of the women’s movements in neo-patriarchal Libya. Oriente Moderno 2018; 98: 225–246.

[17] UNAIDS. Country progress report – Libya, global AIDS monitoring 2020. UNAIDS, 2020, UNAIDS, https://www.unaids.org/en/dataanalysis/knowyourresponse/countryprogressreports/2020countries#L

[18] Elfituri A Kriem F Sliman H , et al. Education against HIV/AIDS. In: Letamo G (ed.) Social and psychological aspects of HIV/AIDS and their ramifications, 2011, pp. 105–124.DOI: 10.5772/23603

[19] Syed Wali P Kumar PGN Karthikeyan R , et al. Knowledge and attitudes of Libyan dental students about HIV/AIDS infection and HIV-positive patients. Dent Med Res 2015; 3: 8–14.

[20] Bugaigis H Tantoush M . Women in the Libyan Job Market: reality and challenges. Friedrich-Ebert-Stiftung and Jusoor Center for Studies and Development, 2017, https://docs.euromedwomen.foundation/files/ermwf-documents/7511_4.6.womeninthelibyanjobmarket-realityandchallenges.pdf

[21] Interagency Coalition on AIDS and Development. HIV/AIDS and gender issues. Ottawa, ON, Canada: Interagency Coalition on AIDS and Development, 2006.

[22] Shawky S Soliman C Kassak KM , et al. HIV surveillance and epidemic profile in the Middle East and North Africa. J Acquir Immune Defic Syndr 2009; 51(Suppl. 3): S83–S95. 19553783 10.1097/QAI.0b013e3181aafd3f PMC3356162

[23] Al-Boom RF . AIDS patients in Libya: we are not criminals! Correspondents, 27 November 2016, https://correspondents.org/2013/11/27/

[24] Rhouma AHHN . Mental health services in Libya. Bjpsych Int 2016; 13: 70–71. 29093908 10.1192/s2056474000001288 PMC5618881

[25] Oraby D . Women living with HIV in the Middle East and North Africa. Lancet Pub Heal 2018; 3(2): e63. 10.1016/S2468-2667(18)30007-0 29422188

[26] Biri E-W Pendleton BF Garland TN . Correlates of men’s attitudes toward women’s roles in Libya. Int J Intercult Rel 1987; 11: 295–312.

[27] El Abani S Pourmehdi M . Gender and educational differences in perception of domestic violence against women among Libyan migrants in Manchester. J Interpers Viol 2018; 36: 2074–2096. 10.1177/0886260518760006 29475425

[28] Abdul-Latif R . Libya status of women survey report, 2013. International Foundation for Electoral Systems, https://www.ifes.org/sites/default/files/libya_status_of_women_survey_report_final2_2.pdf

[29] World Health Organization. Service availability and readiness assessment of the public health facilities in Libya. Libya: Ministry of Health, 2017.

[30] El Issawi F . Women and media: Libyan female journalists from Gaddafi media to post-revolution: case study. Cyber Orient 2014; 80–106

[31] Boller E . ‘There are no women’ the war in Libya in TV news. In: von der Lippe B Ottosen R (ed.) Gendering War and Peace, Gothenburg: Nordicom, 2016, pp. 91–108.

[32] Women’s Human Rights Defenders (WHRD). Status of women human rights defenders in Libya. Women’s Human Rights Defenders, 2017, whrdmena.org

[33] Hyun-kyung K . Arab Spring in Libya – traumatic, yet fruitful in women’s rights. The Korea Times News. Available at: https://m.koreatimes.co.kr/pages/article.asp?newsIdx=240924

[34] Jalova M . Re-place-ing space in Libyan women’s language learning and culture mapping. SHS Web Conf 2017; 33: 00032.

[35] IAGCI. Country policy and information note Libya: Women. London Home Office, 2018, https://www.gov.uk/government/publications/libya-country-policy-and-information-notes

[36] Alzaquzi H Almaghur L Eshagrouni A , et al. Prevalence of high-risk human papillomavirus types 16 and 18 among Libyan women in Tripoli Libya. Libyan J Med Sci 2019; 3: 125–130.

[37] Mirzoyan L Berendes S Jeffery C , et al. New evidence on the HIV epidemic in Libya: why countries must implement prevention programs among people who inject drugs. J Acquire Imm Def Synd 2013; 62: 577–583. 10.1097/QAI.0b013e318284714a 23337363

